# The extent of error-prone replication restart by homologous recombination is controlled by Exo1 and checkpoint proteins

**DOI:** 10.1242/jcs.152678

**Published:** 2014-07-01

**Authors:** Ellen Tsang, Izumi Miyabe, Ismail Iraqui, Jiping Zheng, Sarah A. E. Lambert, Antony M. Carr

**Affiliations:** 1 Genome Damage and Stability Centre, University of Sussex, Brighton, Sussex BN1 9RQ, UK; 2 Institut Curie-Centre National de la Recherche Scientifique, UMR3348, Réponse Cellulaire aux Perturbations de la Réplication, Centre Universitaire, Bat 110, 91405 Orsay, France; 3 Department of Biotechnology, College of Agriculture, No.58 Renmin Avenue, Haikou, Hainan Province 570228, P.R. China

**Keywords:** Checkpoint, Genome instability, Homologous recombination

## Abstract

Genetic instability, a hallmark of cancer, can occur when the replication machinery encounters a barrier. The intra-S-phase checkpoint maintains stalled replication forks in a replication-competent configuration by phosphorylating replisome components and DNA repair proteins to prevent forks from catastrophically collapsing. Here, we report a novel function of the core *Schizosaccharomyces pombe* checkpoint sensor kinase, Rad3 (an ATR orthologue), that is independent of Chk1 and Cds1 (a CHK2 orthologue); Rad3^ATR^ regulates the association of recombination factors with collapsed forks, thus limiting their genetic instability. We further reveal antagonistic roles for Rad3^ATR^ and the 9-1-1 clamp – Rad3^ATR^ restrains MRN- and Exo1-dependent resection, whereas the 9-1-1 complex promotes Exo1 activity. Interestingly, the MRN complex, but not its nuclease activity, promotes resection and the subsequent association of recombination factors at collapsed forks. The biological significance of this regulation is revealed by the observation that Rad3^ATR^ prevents Exo1-dependent genome instability upstream of a collapsed fork without affecting the efficiency of recombination-mediated replication restart. We propose that the interplay between Rad3^ATR^ and the 9-1-1 clamp functions to fine-tune the balance between the need for the recovery of replication through recombination and the risk of increased genome instability.

## INTRODUCTION

Replicative stress can be caused by a wide variety of situations, including tightly bound protein–DNA complexes, clashes of the replication machinery with other cellular processes (i.e. transcription), the presence of non-canonical DNA structures and nucleotide precursor depletion (Lambert and Carr, 2013a). The intra-S-phase checkpoint acts within S phase and promotes cell survival and genome stability in response to replicative stress (Lindsay et al., 1998; Lopes et al., 2001; Tercero and Diffley, 2001) by stabilising arrested forks. In all organisms studied, the intra-S-phase checkpoint requires the activity of two kinases – a phosphoinositol-3 kinase-like kinase (PIKK), known in metazoans as ATR, that senses replication problems by interacting directly with single-stranded DNA (ssDNA)-binding proteins (Zou and Elledge, 2003) and a downstream checkpoint kinase that is directly activated by ATR through interactions with the mediator protein Claspin (Errico and Costanzo, 2012; Segurado and Tercero, 2009).

In the fission yeast *Schizosaccharomyces pombe*, the ATR homologue is known as Rad3^ATR^ and the downstream effector kinase for the intra-S-phase checkpoint is Cds1^Chk2^ (Lindsay et al., 1998). As is the case in mammalian cells, but not for the budding yeast *Saccharomyces cerevisiae*, the heterotrimeric checkpoint clamp complex, composed of Rad9, Rad1 and Hus1 (9-1-1) is also essential for the intra-S-phase checkpoint (Errico and Costanzo, 2012). Activation of the intra-S-phase checkpoint results in phosphorylation of a wide range of replication proteins and DNA repair proteins (Bailis et al., 2008; Boddy et al., 2000; Chen et al., 2010; De Piccoli et al., 2012; Hu et al., 2012; Miyabe et al., 2009; Segurado and Tercero, 2009; Smolka et al., 2007). The precise details of how these phosphorylation events regulate DNA replication and DNA metabolism to stabilise the arrested fork remain largely obscure. In part, this is because the full range of phosphorylation events have not yet been fully mapped and phenotypically characterised. It is also because, in the absence of the intra-S-phase checkpoint, the DNA structures that are initially present at the replication fork in S phase are processed (Sogo et al., 2002) into different structures [i.e. into ssDNA and double-strand breaks (DSBs) (Sabatinos et al., 2012)]. These can both signal through the G2 DNA damage checkpoint and be repaired in a distinct manner from the original lesion. Further complicating genetic analysis, the DNA damage checkpoint requires the function of many of the same proteins as the intra-S-phase checkpoint, with the exceptions that the effector kinase and mediator proteins are replaced (Carr, 2002; Stracker et al., 2009).

Experimentally, replication stress is often imposed by treating cells with the ribonucleotide reductase inhibitor hydroxyurea to globally inhibit replication. During the subsequent dNTP depletion, the intra-S-phase checkpoint stabilises the slowed-down replication forks in a replication-competent state (Lopes et al., 2001). Here, we will refer to these stabilised structures as ‘stalled’ forks. Stalled forks can resume replication without intervention from additional mechanisms when the blockade is removed. By contrast, hydoxyurea treatment in the absence of the intra-S-phase checkpoint results in replication forks that cannot resume (Sabatinos et al., 2012; Sogo et al., 2002). We refer to these as ‘collapsed’ forks. Fork collapse likely occurs when the activities of the replicative helicase and the replicative polymerases are uncoupled, generating extensive stretches of ssDNA. Collapsed forks can also result from clashes between replisomes and the transcription machinery or tightly DNA-bound protein complexes. It has been reported that collapsed forks are no longer associated with components of the replisome, i.e. the replication machinery is not available for DNA synthesis (Cobb et al., 2003; Cobb et al., 2005; Katou et al., 2003; Lucca et al., 2004). However, this might be a simplification, and the machinery might still be present, but no longer able to resume replication (De Piccoli et al., 2012).

Irrespective of the precise nature of the replication machinery present at collapsed forks, it has been demonstrated that, in the absence of the intra-S-phase checkpoint, the genome of hydroxyurea-treated yeast cells is degraded by nucleases, including Mus81 (Boddy et al., 2000; Froget et al., 2008; Kai et al., 2005) and Exo1 (Cotta-Ramusino et al., 2005; Lopes et al., 2001; Segurado and Diffley, 2008; Sogo et al., 2002), and that this degradation is prevented, at least in part, by the phosphorylation of a range of replication proteins and proteins that process specific DNA structures (Chen et al., 2010; De Piccoli et al., 2012; Hu et al., 2012; Segurado and Tercero, 2009; Smolka et al., 2007). The precise physical consequences of fork collapse at the level of the resulting DNA structure remain largely unclear. It is also not clear whether the phenomenon of fork collapse is a cause or consequence of inappropriate DNA processing. However, once a fork does collapse, the DNA is exposed to recombination events that can potentially lead to genome instability (Alabert et al., 2009; Barlow and Rothstein, 2009; Iraqui et al., 2012; Lambert et al., 2010; Lambert et al., 2005; Lisby et al., 2004; Meister et al., 2005; Mizuno et al., 2009; Myung et al., 2001; Myung and Kolodner, 2002; Segurado and Diffley, 2008).

Nucleotide depletion is only one of many potential barriers to replication. Although avoiding fork collapse is one key function of the intra-S-phase checkpoint, in certain situations the collapse of an arrested replication fork might be preferable to its stabilisation. In other cases, replication fork collapse might be unavoidable; for example, when the replisome is blocked by an inter-strand crosslink there is not likely to be sufficient ssDNA to activate the intra-S-phase checkpoint. Interestingly, loss of the intra-S-phase effector kinase Cds1^Chk2^ in fission yeast increases the resistance of otherwise wild-type cells to treatment with the DNA inter-strand crosslinking agent nitrogen mustard (Lambert et al., 2003). This suggests that the initial activation of the checkpoint effector kinase is detrimental to cell survival in these circumstances (reviewed in Lambert and Carr, 2013a). There is indirect evidence to suggest that the intra-S-phase checkpoint proteins regulate the use of recombination for the subsequent repair or restart of collapsed replication forks (Haghnazari and Heyer, 2004; Pandita et al., 2006; Sørensen et al., 2005). However, the mechanisms by which the checkpoint proteins might facilitate recombination at collapsed replication forks are not clear; for example, checkpoint proteins might function to directly promote recombination or they might favour certain recombination pathways over others (Haghnazari and Heyer, 2004; Kolodner et al., 2002).

**Fig. 1.**
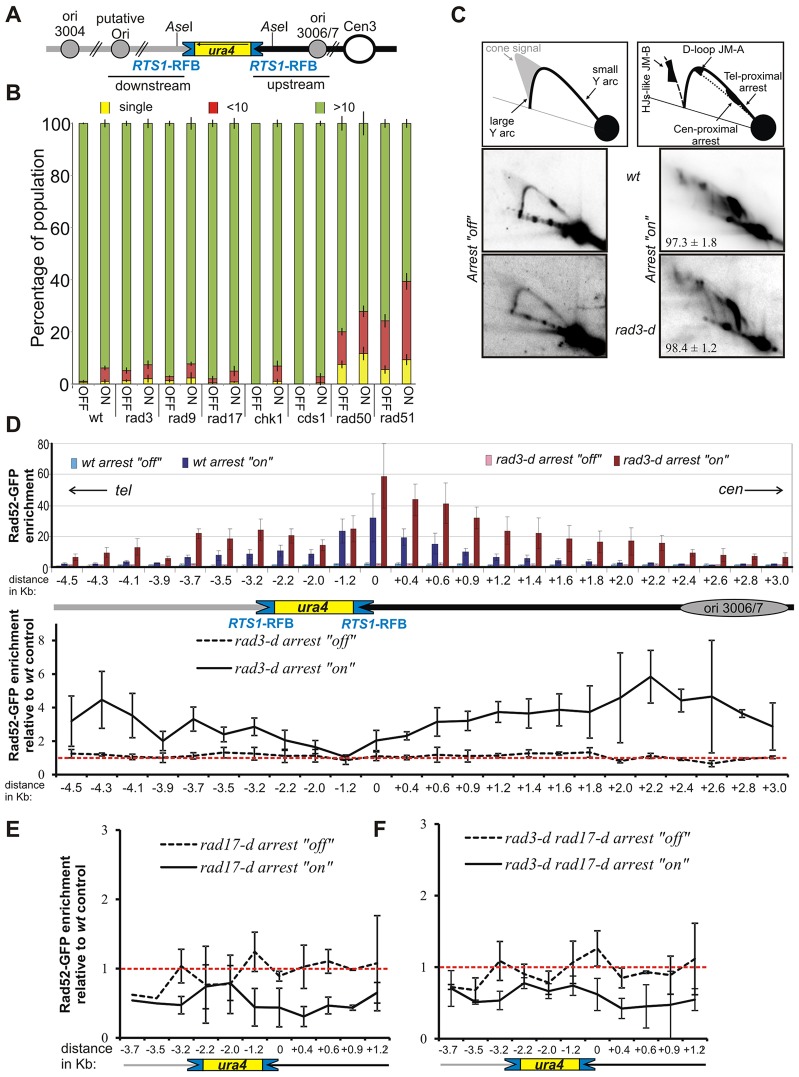
**The checkpoint proteins Rad3^ATR^ and Rad17 regulate the recruitment of Rad52 to the *RuraR* locus.** (A) Schematic representation of the *RuraR* locus. Grey and black lines, telomere- and centromere-proximal sides of the *ura4* gene, respectively; blue boxes, *RTS1*-RFB sequences and their polarity; black arrow indicates the orientation of the *ura4* gene. The nearest replication origin (ori3006/7, grey circles) is located 5 kb *cen*-proximal to *RuraR*. *Ase*I sites are ∼1 kb *cen*-proximal and 0.6 kb *tel*-proximal from *RTS1*. (B) Checkpoint pathways do not affect viability in the *RuraR* system. *RuraR* cells with the indicated genetic backgrounds were grown for 24 hours either with or without thiamine (replication arrest ‘off’ and ‘on’, respectively) and plated onto YE agar plates. The percentage of single cells (unable to divide), micro-colonies of <10 cells (unable to sustain division) and colonies with >10 cells was estimated after 18 hours. The wild-type (wt) control strain contains the native *ura4* locus with no flanking *RTS1* sequences. (C) Lower panels, analysis of replication intermediates by 2DGE of DNA from the indicated strains grown for 24 hours in medium containing or lacking thiamine (fork arrest ‘off’ and ‘on’, respectively). Numbers indicate the percentage of forks arrested by the *RTS1*-RFB (±s.d.). Upper panels, diagrams of replication intermediates within the *Ase*I restriction fragment analysed by 2DGE under the indicated conditions. HJ, Holliday junction; JM-A, joint-molecule A, corresponding to a D-loop intermediate (Lambert et al., 2010); JM-B, joint-molecule B corresponding to recombination intermediates containing HJs (Lambert et al., 2010). (D) Regulation of Rad52 recruitment to *RuraR*. Chromatin immunoprecipitation (ChIP) of Rad52–GFP followed by quantitative PCR (qPCR) was performed on the indicated *RuraR rad52-GFP* strains after 40 hours of growth either with or without thiamine (arrest ‘off’ and ‘on’, respectively). Cells containing the *RuraR* locus in a checkpoint-proficient (*wt*) background were analysed, alongside an isogenic strain harbouring the *rad3-d* alleles. The schematic is as described for A. Upper panel, data show the mean±s.e.m. (three independent experiments). Lower panel, enrichment in the *rad3-d* strain relative to the wild-type strain. Data show the mean±95% confidence intervals (CI) (three independent experiments). When the errors bars do not overlap the red dotted line (relative enrichment of 1), the level of Rad52 enrichment observed in the *rad3-d* strain is significantly different from the that of the wild-type strain (*P*<0.05). Numbers indicate the distance (kb) from the *RTS1*-RFB on the telomere (−) and centromere-proximal (+) sides, with the closest RFB to *ori3006/7* being used as referential (0). (E,F) Rad52–GFP enrichment in the *rad17-d* strain (E) and the double mutant *rad3-d rad17-d* (F) relative to the wild-type strain, as described for D. Data show the mean±95% CI (two to three independent experiments).

**Fig. 2.**
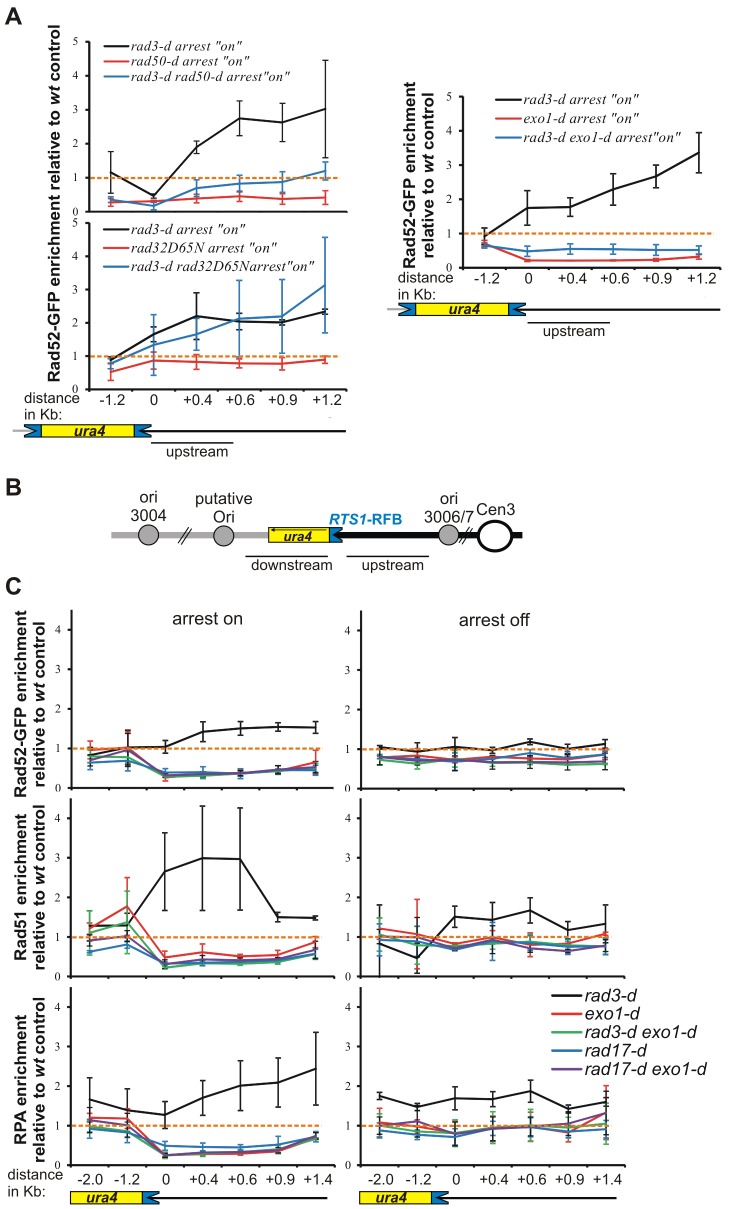
**Rad3^ATR^ and Rad17 regulate Exo1-dependent recruitment of ssDNA-binding proteins at collapsed forks.** (A) Rad52–GFP enrichment relative to wild-type control (*wt*) at the *RuraR* locus in the indicated strains, as described for Fig. 1D. Data show the mean±95% CI (three independent experiments). Schematics are as described for Fig. 1A. (B) Schematic representation of the *uraR* locus, as described for Fig. 1A. (C) Relative enrichment of Rad52–GFP (upper panels), Rad51 (middle panels) and RPA (lower panels) relative to the wild-type control (*wt*) for indicated strains, as described for Fig. 1D. ChIP followed by qPCR was performed on the indicated *uraR* strains after 40 hours of growth either with or without thiamine (arrest ‘off’ and ‘on’, respectively). Orange dotted lines, relative enrichment of 1. Data show the mean±95% CI (three independent experiments).

The activation of the intra-S-phase checkpoint and its components has been largely characterised in response to acute replication stress (such as hydroxyurea treatment), whereas the cellular response to chronic and endogenous replication stress is less well characterised, despite the fact that this represents the main source of replication-induced genetic instability in pre-neoplastic lesions (Bester et al., 2011; Halazonetis et al., 2008). In this report, we have therefore used an established replication fork barrier (RFB) that induces a local and chronic replication stress to explore the ATR-dependent checkpoint response. The RFB we have exploited is the *RTS1* sequence in fission yeast. *RTS1* is a well-characterised polar RFB that requires a sequence specific Myb-domain DNA-binding protein, Rtf1, for its function (Lambert and Carr, 2005; Lambert et al., 2010). In wild-type *S. pombe* cells, *RTS1* resides close to the mating-type (*mat*) locus and, when bound by Rtf1, functions to block replication forks passing in one direction while allowing them to pass unhindered in the opposite direction (Eydmann et al., 2008). Although *RTS1*:Rtf1 is not directly involved in mating-type switching, its barrier activity facilitates switching by preventing inappropriate forks moving through the switching region in the wrong direction (Codlin and Dalgaard, 2003; Dalgaard and Klar, 2001; Lee et al., 2004). In our experimental systems, either one or two copies of the 850-bp *RTS1* sequence are positioned at the *ura4* locus (Fig. 1A; Fig. 2B) and *rtf1^+^*, which is essential for *RTS1* RFB activity, is under the control of a thiamine-repressible *nmt* promoter. Upon induction of *rtf1*^+^ transcription, forks arrest and rapidly collapse (Lambert et al., 2005). Recombination proteins are required for fork restart (Lambert et al., 2010), which occurs within 20 minutes (unpublished data). There is no cell cycle arrest resulting from this DNA processing (supplementary material Fig. S1) (Lambert et al., 2005), consistent with there being sufficient time within the normal cell cycle to restart the collapsed forks by homologous recombination.

## RESULTS

### Checkpoint genes are not essential in the RuraR system

Using a construct where two *RTS1* sequences are integrated at the *ura4* locus as inverted repeats flanking *ura4*^+^ (*RuraR*), we have previously demonstrated that, when forks arrest at *RTS1*, they are subject to recombination-mediated restart (Lambert et al., 2005). Greater than 94% of forks arrest when they encounter the RFB, and the vast majority of these restart correctly (Lambert et al., 2010; Mizuno et al., 2013) within 20 minutes and complete replication. In the *RuraR* system (Fig. 1A), cell viability is impaired when the recombination pathway, but not the Rad3^ATR^ checkpoint protein, is compromised (Lambert et al., 2005) and no cell cycle delay is observed when the *RTS1*-RFB is induced (Lambert et al., 2005; supplementary material Fig. S1). To extend this observation, we crossed the *RuraR* locus into backgrounds null for *cds1*, *chk1* (downstream effector kinases), *rad17* (checkpoint clamp loader) and *rad9* (9-1-1 checkpoint clamp subunit). We used a micro-colony assay to establish the percentage of cells able to form colonies of greater than ten cells when replication arrest was either induced or not induced. We confirmed that (unlike recombination-defective *RuraR* control strains) viability was not significantly affected by checkpoint loss; *rad3*-, *rad9*-, *rad17*-, *cds1*- and *chk1*-null *RuraR* cells were all able to form micro-colonies as efficiently as the checkpoint-proficient *RuraR* strain (Fig. 1B). In order to rule out the possibility that checkpoint mutants are defective for *RTS1*-dependent RFB activity, we assayed replication intermediates in *rad3*-null and *rad3*^+^ control strains grown both with and without thiamine. The extent of fork pausing at *RuraR* in *rad3*-null cells (98.4%±1.2 of arrested forks, ±s.d.) was comparable to that observed for wild-type cells (97.3%±1.8) (Fig. 1C).

Thus, replication completion upon activation of the *RTS1*-RFB does not require checkpoint pathways or cell cycle delay. This is in contrast to acute replication stresses caused by agents such as hydroxyurea. These circumstances thus allow us to separate the known roles of the intra-S-phase and replication checkpoints – namely, promoting replication resumption by preventing fork collapse and delaying the cell cycle, respectively – from any potential functions in either regulating DNA metabolism at a collapsed fork or in regulating the ensuing choice of homologous recombination pathway. We thus set out to examine the recruitment of homologous recombination proteins to the collapsed fork at *RTS1* and to observe potential changes to aberrant recombination outcomes caused by the loss of checkpoint proteins.

### Regulation of Rad52 recruitment by checkpoint proteins

We demonstrated previously that induction of replication fork arrest at *RTS1* leads to the recruitment of Rad52 to the *RuraR* locus (Lambert et al., 2005). To establish whether the extent of recruitment is subject to checkpoint regulation, we performed chromatin immunoprecipitation (ChIP) analyses against Rad52–GFP in checkpoint-mutated *RuraR* strains (Fig. 1D) following growth for 40 hours either with thiamine (arrest ‘off’) or without thiamine (arrest ‘on’). Transcription is induced by the *nmt41* promoter ∼16 hours after thiamine removal (Maundrell, 1993). Thus, at 40 hours in the absence of thiamine, the culture is in a steady state, where forks arrest at *RuraR* during each S phase and replication of the locus is reliant on homologous-recombination-dependent fork restart. Consistent with this, we have demonstrated previously that fork arrest at *RuraR* is not detectable at 12 hours following thiamine removal, but occurs with similar efficiency at both 24 and 48 hours after thiamine removal (compare with figure 1 in Lambert et al., 2005).

In the wild-type strain background, ‘arrest on’ conditions resulted in Rad52 recruitment at, and immediately flanking, the *RuraR* locus (Fig. 1D, top panel, blue bars). As expected, enrichment was most prevalent at the right-hand [centromere (*cen*)-proximal] barrier, because the direction of replication fork movement is from right to left (Mizuno et al., 2013). Enrichment persisted for ∼2 kb *cen*-proximal to the *RTS1* sequence. A second less-prevalent region of enrichment extended ∼1 kb from the left-hand barrier towards the telomere (*tel*). In the *rad3*^ATR^-null background, Rad52 recruitment was significantly increased, both at *RTS1* sequences and in the flanking regions, and spread further behind the fork arrest site, >3 kb *cen*-proximal to the *RTS1* sequence (Fig. 1D, top panel, red bars). To better visualise the role of Rad3^ATR^ in regulating Rad52 association at the *RuraR* locus, Rad52 enrichment in *rad3-d* cells was calculated relative to that observed for the wild-type control (Fig. 1D, bottom panel). This confirmed that Rad52 was up to six times enriched at both *tel*- and *cen*-proximal regions flanking the *RTS1*-RFB in *rad3-d* cells when compared with its enrichment in *rad3*^+^ cells.

In contrast to the higher recruitment of Rad52 in the *rad3*-null strain, in the strains null for *rad17* (clamp loader, Fig. 1E) and *rad9* (9-1-1 complex subunit, supplementary material Fig. S2A,B), Rad52 recruitment was significantly reduced by approximately twofold at *RTS1* sequences and at both the *tel*- and *cen*-proximal flanking sequences relative to its recruitment in wild-type cells. We also assessed the enrichment of Rad52 at the *RuraR* locus in the double *rad3*^ATR^-*rad17*-null mutant. This mutant showed a Rad52 recruitment profile similar to that of *rad17*-null cells (Fig. 1E,F), indicating that the effect of losing Rad3^ATR^ function requires a functional Rad17/9-1-1 clamp. Interestingly, neither the *chk1*-null nor the *cds1^Chk2^*-null strains showed a reproducible change in Rad52 recruitment when compared with that of wild-type cells (supplementary material Fig. S2C), a result consistent with there being no evidence for checkpoint-dependent cell cycle arrest (supplementary material Fig. S1). These data show that the checkpoint sensor Rad3^ATR^ prevents the extensive recruitment of Rad52 upstream of arrested forks, whereas 9-1-1 clamp loading, dependent on the sensor Rad17, promotes such Rad52 recruitment. Moreover, the function of checkpoint sensors in regulating Rad52 association at collapsed forks is independent of the downstream effector kinases Chk1 and Cds1^Chk2^. To address whether the regulation of Rad52 association by the checkpoint proteins is related to the formation of ssDNA at blocked forks, we next investigated the role of nuclease activities in Rad52 association.

### Regulation of Rad52 recruitment by MRN and Exo1

In response to a DNA DSB, the MRN complex functions to initiate resection (Mimitou and Symington, 2008; Raynard et al., 2008; Symington, 2002). The subsequent generation of ssDNA is largely Exo1-dependent, with a later contribution from Rqh1^RecQ^ and Dna2. When a fork collapses at *RTS1* there is no DSB formation (Mizuno et al., 2009), and homologous-recombination-dependent replication restart occurs from a single-stranded gap (Lambert et al., 2010). To establish whether MRN or the Exo1 nuclease participate in DNA metabolism at *RTS1*-induced collapsed forks, we investigated the involvement of MRN and Exo1 in Rad52 loading. When the replication fork arrest was induced in a strain null for *rad50* (an MRN component), Rad52 recruitment to *RuraR* was reduced relative to that of the wild-type strain (Fig. 2A, left panels). Similarly, the increased recruitment of Rad52 in the *rad3*-null background was also reduced. In contrast to *rad50*-null cells, cells carrying the *rad32^mre11^-D65N* nuclease-deficient allele (Hartsuiker et al., 2009) did not display changes in Rad52 enrichment levels in either the *rad3^+^* or *rad3*-null strains (Fig. 2A). Thus, an intact MRN complex, but not the nuclease activity of the MRN subunit Rad32^Mre11^, is required for Rad52 recruitment, and the increased loading observed in a *rad3*-null background is similarly MRN-dependent.

Exo1 has been implicated with Mre11 in the resection of DSBs to generate ssDNA (Mimitou and Symington, 2008; Moreau et al., 1999; Moreau et al., 2001; Tsubouchi and Ogawa, 2000; Zhu et al., 2008). Exo1 has been reported to be negatively regulated by Mec1^ATR^ and Rad53^Chk2^ in the generation of ssDNA at uncapped telomeres in budding yeast (Jia et al., 2004; Morin et al., 2008; Zubko et al., 2004). Again in *S. cerevisiae*, during DNA replication, Exo1 is proposed to travel with active replication forks and is known to participate in the instability of stalled forks in the absence of regulation by Rad53^Chk2^ (Cotta-Ramusino et al., 2005; Segurado and Diffley, 2008). In our analysis, an *exo1*-null strain showed a significant decrease in Rad52 recruitment to *RuraR*, both in the *rad3*^+^ background and in combination with a *rad3*-null mutant (Fig. 2A, right panel). Thus, at collapsed forks, Exo1 is required for normal Rad52 recruitment to single-stranded gaps in checkpoint-proficient cells, and it mediates the extensive Rad52 loading observed in *rad3*-null cells.

### RPA, Rad52 and Rad51 are recruited upstream of the site of fork arrest

Rad52 is known to bind to replication protein A (RPA)-coated ssDNA, replacing the RPA to initiate homologous recombination by nucleating Rad51 filaments (Krejci et al., 2012). Thus, Rad52 recruitment immediately upstream of the site of fork collapse is strongly indicative of DNA processing. We next verified that both Rpa1 and Rad51 were also recruited with similar profiles. To clearly distinguish the recruitment of proteins either upstream or downstream of the collapsed fork, we used the *uraR* construct in which a single *RTS1* barrier is located *cen*-proximal to *ura4^+^* (Lambert et al., 2010) (Fig. 2B). The use of the *uraR* locus simplifies the analysis because the *RuraR* locus, in addition to recombination-mediated fork restart at the site of fork collapse, is also subject to recombination events dependent on a template switch between the two inverted *RTS1* repeats (Lambert et al., 2010). Such events could influence the location of Rad52 recruitment in the vicinity of the *RTS1*-RFB (Lambert et al., 2010).

Similar to the increased Rad52 association observed at *RuraR*, the binding of Rad52, Rad51 and RPA were all increased at *uraR* in *rad3*-null cells when compared with that of *rad3*^+^ controls, particularly upstream of the site of fork arrest (Fig. 2C). The association of these ssDNA-binding proteins with *uraR* was also dependent on Rad17 and Exo1, as observed for the *RuraR* construct. Moreover, the double *rad17*-*exo1*-null mutant showed a similar reduction in Rad52 binding upstream of the *RTS1*-RFB to that observed in each single mutant, suggesting that Rad17 and Exo1 act in the same pathway of Rad52 recruitment at arrested forks (Fig. 2C). These data are consistent with the idea that Rad3^ATR^ limits extensive Exo1-dependent resection behind collapsed forks, whereas the PCNA-like 9-1-1 complex promotes it.

### Checkpoint proteins do not influence template exchange at *RuraR*

Taken together, our data strongly imply that DNA is resected upstream of a collapsed replication fork in a manner that is dependent on Rad17, the 9-1-1 complex, MRN and Exo1 and is attenuated by the activity of Rad3^ATR^. We have reported previously that the majority of arrested forks rapidly restart correctly by homologous recombination and complete replication, but that, in the *RuraR* system, 2–5% of cells in each generation undergo inappropriate template exchange with the nearby inverted repeat (Lambert et al., 2010; Lambert et al., 2005; Mizuno et al., 2009). This results in intra-chromosomal recombination, leading to either inversion of the *ura4* gene or the formation of an acentric and dicentric chromosome (supplementary material Fig. S3). Both of these events can be monitored by Southern blotting and pulse-field gel analysis and, upon fork arrest, the frequency of the rearrangement-specific band increases with each generation. We thus analysed wild-type, checkpoint-null and *exo1*-null *RuraR* cultures at T_0_ (fork arrest ‘off’) and after 48 hours of growth without thiamine, T_48_ (fork arrest ‘on’), for fork-arrest-induced recombination intermediates [using two-dimensional gel electrophoresis (2DGE); supplementary material Fig. S3B,C] and for both recombination outcomes – acentric chromosome formation (supplementary material Fig. S3D) and *ura4*^+^ inversion (supplementary material Fig. S3E).

In *rad3*-, *rad17*- and *exo1*-null mutant backgrounds, recombination intermediates (D loops and structures containing Holliday junctions) occurred at a frequency equivalent to that of the wild-type control, and the recombination outcomes were unchanged. Thus, the perturbation in resection and in recombination protein loading seen in the checkpoint mutants or the *exo1* mutant did not significantly influence the amount of homologous-recombination-dependent replication restart after fork collapse or the types of deleterious intra-chromosomal recombination events that occur due to faulty template exchange between *RTS1* sequences at *RuraR*. Our data therefore suggest that a limited amount of recombination factors are sufficient to promote replication restart by template exchange.

### Extensive resection behind the fork results in increased genetic instability

**Fig. 3.**
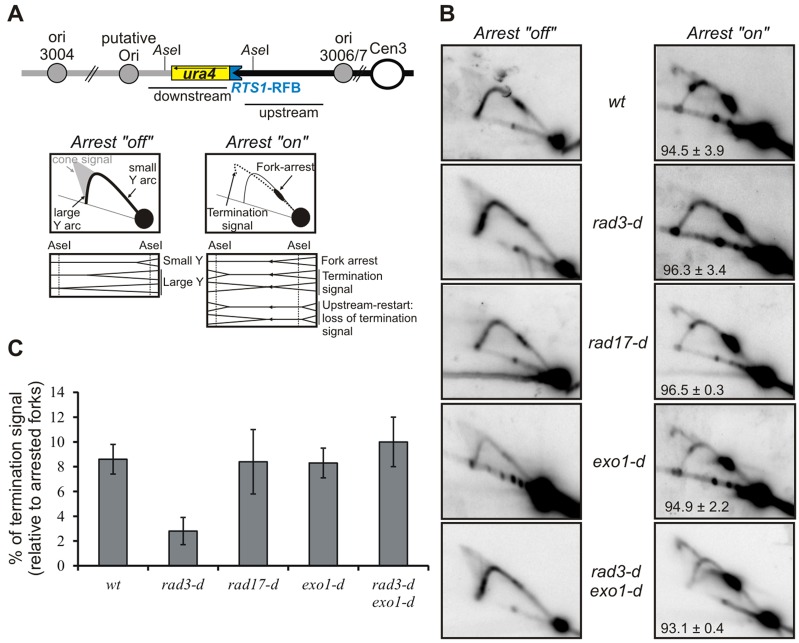
**Exo1-dependent fork resection is regulated by Rad3^ATR^ and Rad17.** (A) Schematic representation of the *uraR* locus, as presented in Fig. 1A. Panels show diagrams of replication intermediates within the *Ase*1 restriction fragment as analysed by 2DGE under the indicated conditions. (B) Analysis of replication intermediates by 2DGE from the indicated strains after growth for 24 hours in medium with or without thiamine (fork arrest ‘off’ and ‘on’, respectively). *wt*, wild-type control. Numbers indicate the percentage of forks arrested by the *RTS1*-RFB (±s.d.) (C) Quantification of the termination signal from B in the indicated strains. Data show the mean±s.d. (three independent experiments).

Our data are consistent with a model whereby the extent of resection upstream of the collapsed fork is not rate-limiting for fork restart. However, extensive resection, such as that seen in the *rad3*-null mutant background, implies that restart must frequently occur a significant distance upstream of the point of the original fork collapse. To establish whether this is the case, we visualised the converging fork signal (Fig. 3A) by 2DGE analysis. In a wild-type background, ∼8% of the replication intermediates represented converging forks that we assume arise when an incoming replisome from the *tel*-proximal side approaches the arrested fork structure close to, or within, *RTS1*. In *rad17*- or *exo1*-null backgrounds, this signal remained constant. However, in *rad3*-null cells, where resection is proposed to be extensive, the signal was reduced by more than threefold (Fig. 3B,C). This is consistent with the expectation that resection beyond the restriction site *cen*-proximal to *RTS1* (that defines the fragment being analysed by 2DGE, see the right-hand diagram in Fig. 3A) would result in a restart event upstream of the initial point of fork collapse and thus loss of the converging fork signal within the restriction fragment analysed by 2DGE. Concomitant loss of *exo1* in the *rad3*-null mutant restored the converging fork signal to the wild-type level, consistent with the loss of the termination signal being a consequence of extensive resection.

**Fig. 4.**
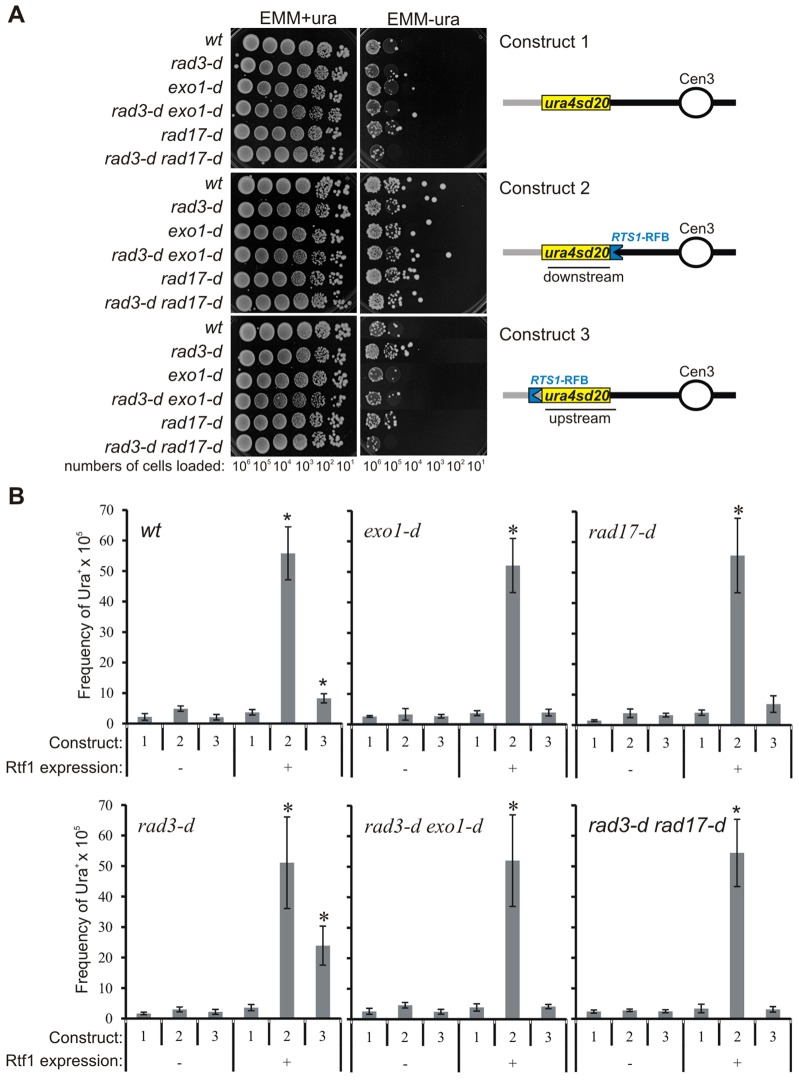
**Increased replication slippage correlates with increased RPA recruitment.** (A) Assays of fork-arrest-induced replication slippage. The *ura4-sd20* allele contains a duplication of 20 bp flanked by 5 bp of microhomology and is non-functional – cells are thus auxotroph for uracil. Upon activation of the *RTS1*-RFB, the recombination-dependent restart of DNA synthesis is error prone and liable to replication slippage, leading to the deletion of the duplication and, thus, the restoration of a functional *ura4* gene – cells are thus prototroph for uracil. Schematics are as described for Fig. 1A. The *ura4-sd20* allele (yellow) is either located downstream (construct 2) or upstream (construct 3) of the *RTS1*-RFB. Construct 1 is the control (without any *RTS1*-RFB) that is used to score the spontaneous frequency of replication slippage for each genetic background when Rtf1 is expressed. Serial dilutions of cells from the indicated strains were spotted onto medium containing or lacking uracil after growth in medium without thiamine (Rtf1 being always expressed). *wt*, wild-type control. (B) Frequency of ura^+^ reversion in the indicated strains and constructs when Rtf1 is expressed (+, in medium containing no thiamine) or not (−, in medium containing thiamine). Data show the mean±95% CI (at least three independent experiments). Statistical significance was detected by using the nonparametric Mann-Whitney U test. The asterisk (*) indicates a significant difference in the frequency of replication slippage upon activation of the *RTS1*-RFB (construct 2 or 3, Rtf1^+^) compared with the frequency observed in the strain containing no *RTS1*-RFB upon Rtf1 expression (construct 1, Rtf1^+^).

The restart of the collapsed fork upstream of the initial site of arrest would result in more DNA being replicated by the restarted replication machine. We have shown previously that restarted replication forks are prone to replication slippage at sites of microhomology (Iraqui et al., 2012). Using an assay in which replication slippage removes a short direct repeat from the *ura4-sd20* allele, we therefore tested for evidence that the region upstream of the blocked fork is more susceptible to such slippage errors when resection is extensive (Fig. 4). First, the spontaneous level of replication slippage (irrespective of whether Rtf1 was overexpressed or not) was similar in all the genetic backgrounds tested (*RTS1*-RFB is absent; Fig. 4, see construct 1). Second, fork arrest led to an equivalent increase in replication slippage downstream of the *RTS1*-RFB during replication restart in all genetic backgrounds (Fig. 4A, see construct 2). This observation confirmed that, whatever the level of recombination factors associated with the collapsed replication fork, replication restart occurred efficiently. Third, when the marker gene was placed upstream of the *RTS1*-RFB, fork arrest in checkpoint-proficient cells led to a threefold increase in replication slippage upstream of the site of fork arrest (*P*<1.7×10^−5^) (Fig. 4, see construct 3). This is consistent with occasional leading-strand degradation and subsequent re-synthesis by a homologous-recombination-restarted replication fork upstream of the initial site of fork arrest (Iraqui et al., 2012).

The induction of replication slippage upstream of the site of replication arrest is Exo1-dependent (*P*<0.0002). A 1.9-fold induction of replication slippage was observed (Fig. 4B) in *rad17*-null mutant cells, although this was not statistically significant (*P*>0.05). By contrast, fork arrest led to an 8.7-fold increase in replication slippage upstream of the arrest site in the *rad3*-null mutant (*P*<9.8×10^−5^), corresponding to a 2.9 times higher level when compared with that of the *rad3*^+^ strain (*P*<1.6×10^−5^). Replication slippage occurring upstream of the *RTS1*-RFB in *rad3*-null cells was dependent on both Exo1 and Rad17 (*P*<8.2×10^−6^). These data strongly support our model that efficient homologous-recombination-dependent restart does not require checkpoint activation, but frequently occurs upstream of the site of initial arrest when resection is extensive.

**Fig. 5.**
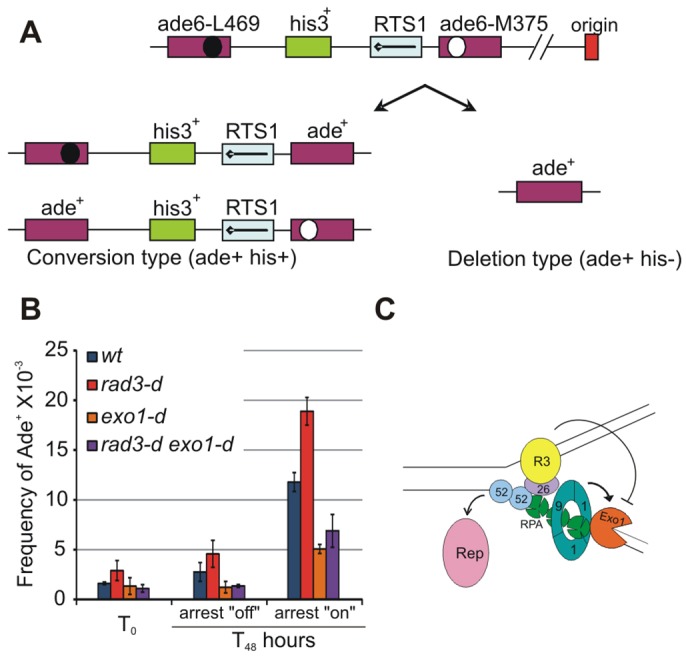
**Rad3^ATR^ regulates *ade6* recombination in an Exo1-dependent manner.** (A) Schematic representation of the *ade6* recombination system. Recombination between the *ade6* heteroalleles can occur by conversion (lower left) or deletion (lower right) pathways. White and black circles represent the mutations in the *ade6* open reading frames. (B) *ade6* recombination frequency was scored in the indicated strains following 48 hours of growth either with or without thiamine (fork arrest ‘off’ and ‘on’, respectively). Cells were plated onto adenine-deficient medium containing thiamine at baseline (T0). *wt*, wild-type control. Data show the mean±s.d. (three independent experiments). (C) Simplified schematic indicating that Rad3^ATR^, Rad26^ATRIP^ (R3, R26) and the 9-1-1 complex regulate Exo1 to reveal ssDNA, which associates with RPA and recombination proteins [e.g. Rad52 (52)] when the replisome (Rep) is no longer competent.

It can also be predicted that the generation of ssDNA behind the collapsed fork would increase opportunities for homologous recombination to occur erroneously upstream of the site of the initial fork arrest. To establish whether the increased DNA processing behind the fork increased non-allelic homologous recombination in this region, we turned to a direct repeat recombination assay (Ahn et al., 2005). It is proposed that recombination between the direct repeats requires nuclease activities to resect nascent strands until a homologous region is exposed as ssDNA (Sun et al., 2008). In this system, two *ade6* heteroalleles are positioned as direct repeats that flank a *his3^+^* marker and a single *RTS1* barrier (Fig. 5A). Replication is predicted to run from right to left at this locus (Heichinger et al., 2006) and, consistent with this, it has been demonstrated that the *RTS1* barrier orientation must arrest right-to-left forks to significantly elevate recombination rates (Ahn et al., 2005).

Ahn and colleagues assayed recombination in the presence of constitutive *rtf1* expression (Ahn et al., 2005). In order to regulate fork arrest, we combined a thiamine-repressible *nmt41-rtf1* allele with their *ade6*-heteroallele locus and scored *ade6* recombination in the wild-type, *rad3*- and *exo1*-null backgrounds at T_0_ (no induced arrest) and after 48 hours either with (arrest ‘off’) or without (arrest ‘on’) thiamine (Fig. 5B). It should be noted that, under the ‘arrest off’ conditions (+ thiamine), the cells retain a low, but significant, level of fork arrest (Lambert et al., 2010; Lambert et al., 2005), and thus these conditions do not fully reflect recombination in the complete absence of arrest. Nonetheless, *rad3*-null mutants in ‘arrest on’ conditions showed elevated levels of recombination (*P*≤0.0019) when compared with those of *rad3*^+^ cells (18.9 versus 11.79 recombinants per 10^3^ cells), and *exo1*-null cells show significantly reduced levels (*P*≤0.0004) of recombination (5.06 versus 11.79 recombinants per 10^3^ cells). Importantly, concomitant deletion of *exo1* in the *rad3*-null background reduced the amount of recombination in the ‘arrest on’ conditions to levels approaching those of the *exo1*-null single mutant (6.88 versus 5.06 recombinants per 10^3^ cells). These data suggest that *RTS1*-induced *ade6* heteroallele recombination is suppressed by Rad3^ATR^ and promoted by Exo1 activity, and that Rad3^ATR^ is inhibiting Exo1-dependent recombination.

## DISCUSSION

The role of the intra-S-phase checkpoint in maintaining arrested replication forks in a replication-competent state is well documented, and the underlying mechanisms are beginning to be unravelled. In this report, we identify a new function for the intra-S-phase checkpoint at collapsed replication forks. Specifically, we show that the recruitment of RPA, Rad52 and Rad51 to the site of a collapsed fork is distinctively controlled by Rad3^ATR^ and the 9-1-1 checkpoint clamp, through the coordination of Exo1- and MRN-dependent resection (Fig. 5C). This checkpoint regulation of DNA processing acts to limit the extent of local replication errors that occur as a consequence of homologous-recombination-dependent replication restart. Moreover, our work reveals a role for the checkpoint sensors, independent of the downstream kinases, in limiting replication-induced genome instability in response to a chronic replication stress, thus contrasting with the classical analysis of checkpoint activation in response to acute replication stress.

### The role of checkpoint proteins at *RTS1*-blocked replication forks

An active replisome moves with the fork and closely couples DNA synthesis to the activity of the replicative helicase (Errico and Costanzo, 2012). The current model is that, if polymerisation is perturbed, the helicase initially moves ahead of the polymerases to expose an additional ∼100 bp of ssDNA (Sogo et al., 2002). This promotes the stimulation of Rad3^ATR^ and local activation of the intra-S-phase checkpoint. The checkpoint kinases (in *S. pombe*, Rad3^ATR^ and Cds1^Chk2^) subsequently phosphorylate a range of replication and repair proteins. This protects the fork from collapse and retains the replisome in an active conformation (De Piccoli et al., 2012) while, at the same time, arresting cell cycle progression. By contrast, if helicase activity, as opposed to polymerisation, is perturbed, the initial exposure of ssDNA does not occur, the intra-S-phase checkpoint is not activated, the replisome cannot be held in an active conformation and the fork will collapse (Lambert and Carr, 2013a).

By analogy with the *Escherichia coli* Tus-*ter* site-specific RFB (Bastia et al., 2008) and the Reb1-dependent barrier at the *S. pombe* rDNA locus (Biswas and Bastia, 2008), we speculate that forks arrest at *RTS1* because the replicative helicase is directly inhibited by the *RTS1*-associated proteins. Thus, because the helicase cannot move ahead of the polymerases, ssDNA is not formed, Rad3^ATR^ is not activated, and the fork collapses. By analysing the association of ssDNA-binding proteins with a specific collapsed replication fork, we have been able to show that the Rad3^ATR^ checkpoint is locally activated by fork collapse to ultimately control the activity of subsequent DNA processing events. Because forks arrested at *RTS1* do not require the intra-S-phase checkpoint for their restart, we have been able to use our model systems to specifically examine the processing of DNA at the site of fork collapse, independently of the consequences of replisome stabilisation. Our data show that Rad3^ATR^-dependent regulation of Exo1-dependent resection results in inappropriate DNA processing of the collapsed fork, but that this does not prevent homologous-recombination-dependent replication restart.

Previous work has identified Exo1 as a significant target of the intra-S-phase checkpoint when ATR is activated to stabilise intact replisomes; in *S. cerevisiae*, Rad53^Chk2^ prevents Exo1-dependent replication fork breakdown in response to global replication stress (Cotta-Ramusino et al., 2005; Segurado and Diffley, 2008), and it has also been suggested that Rad53^Chk2^ phosphorylates and regulates Exo1 at uncapped telomeres (Morin et al., 2008). In mammalian cells, Exo1 has been shown to be phosphorylated at 12 sites, of which three are induced by hydroxyurea treatment in an ATR-dependent manner (Bolderson et al., 2010; El-Shemerly et al., 2008). Thus, our identification of Exo1 as a key target of the ATR pathway at collapsed forks, as well as when forks are being stabilised, emphasises the importance of regulating this nuclease.

Mechanistically, we show that the PCNA-like 9-1-1 checkpoint clamp acts to promote MRN- and Exo1-dependent resection of DNA to extend a region of ssDNA upstream of the collapsed replication fork. This function for the clamp loader and clamp axis of the checkpoint is regulated by Rad3^ATR^, but not by Chk1 or Cds1^Chk2^. Thus, in the absence of Rad3^ATR^ function and the presence of a loaded 9-1-1 complex, repair-protein recruitment is likely to be increased. Unfortunately, we have been unable to generate reagents that are suitable for ChIP of Exo1. However, the likely explanation is that Rad3^ATR^ directly phosphorylates the clamp loader, clamp subunits and/or specific repair proteins recruited by the clamp (candidates include Mre11 and Exo1) to restrict resection. Such a mode of regulation would be fully consistent with the multiple phosphorylation events reported for these proteins.

### Checkpoint regulation of recombination-protein recruitment contributes to genome stability

Replication stress underlies a significant proportion of the genomic instability observed in model organisms and in cancer cells (Carr and Lambert, 2013; Lambert and Carr, 2013b; Segurado and Tercero, 2009). The intra-S-phase checkpoint is essential for maintaining the integrity of replication forks in the presence of such stress (Errico and Costanzo, 2012). Loss of the ability to maintain the replication-competent state of arrested or paused replication forks leads to their collapse, an event that has been linked to increased genome rearrangements in *S. cerevisiae* (Cha and Kleckner, 2002; Kaochar et al., 2010; Myung et al., 2001; Myung and Kolodner, 2002) and the expression of fragile sites in humans (Brown and Baltimore, 2003; Casper et al., 2002; Durkin et al., 2006). We have reported previously that, when a fork collapses, replication restart occurs through a ssDNA intermediate, not from a DSB (Mizuno et al., 2009). We found that fork restart is highly efficient, but is prone to non-allelic homologous recombination (NAHR), i.e. it has a ∼1–3% chance of restarting at the wrong place if a homologous sequence is nearby (Lambert and Carr, 2005; Lambert et al., 2010). We also demonstrated that, once restarted correctly, the restarted replication machinery is prone to replication slippage at sites of microhomology (Iraqui et al., 2012) or to performing a U-turn at closely spaced inverted repeats (Mizuno et al., 2013).

As a consequence of fork collapse and restart, NAHR (associated with the restart event) and replication slippage (associated with the restarted replisome) provide mechanisms for the genomic instability associated with replication stress. We examined the effects of the Rad3^ATR^ checkpoint on these mechanisms of genome instability that are specifically related to collapsed forks, as opposed to stalled forks, and their subsequent resumption of replication. Somewhat to our surprise, we found that recombination-mediated fork restart was independent of the checkpoint, and the frequency of the associated NAHR was unchanged. These data suggest that even limited association of recombination factors at collapsed replication forks is sufficient to ensure their efficient restart. However, the extensive resection we observed in the *rad3*-null mutant prompted us to explore whether this additional DNA processing resulted in more extensive genetic instability associated with the error-prone nature of the restarted replication fork and intra- or inter-sister-chromatid homologous recombination.

We observed that the extent of resection and subsequent recombination protein recruitment correlated directly with the promotion (*rad3* null) or suppression (*rad17* null) of inter- or intra-sister homologous recombination. These data implicate Rad3^ATR^ in limiting genome instability by regulating DNA metabolism and, thus, the activity of homologous recombination behind collapsed replication forks. We also observed a correlation between replication slippage after restart and the extent of resection. These data are entirely consistent with increased resection resulting in a larger region of DNA being replicated by an error-prone restarted fork. Thus, a function of Rad3^ATR^ is to limit the amount of DNA replicated by the restarted fork, which in turn reduces the likelihood of associated genetic instability.

### Conclusions

It has become clear that the intra-S-phase checkpoint, acting through Rad3^ATR^ and Cds1^Chk2^ in *S. pombe*, Mec1^ATR^ and Rad53^Chk2^ in *S. cerevisiae* or ATR and Chk1 in human cells, prevents replication forks collapsing catastrophically, in part by phosphorylating replisome components and specific proteins affecting DNA metabolism, such as Exo1 (reviewed in Errico and Costanzo, 2012; Segurado and Tercero, 2009). A recent report also showed that moderate replication stress in ATR-depleted mammalian cells results in MRN-dependent ssDNA accumulation, the chromatin association of checkpoint sensors and the expression of common fragile sites (Koundrioukoff et al., 2013). Here, using a site-specific replication-arrest system, we have dissected the role of Rad3^ATR^ in fork stabilisation and checkpoint activation away from the roles it plays after the fork has collapsed. We reveal a subtle role for the core checkpoint kinase, ATR, and the 9-1-1 clamp in regulating recombination protein association at collapsed forks (Fig. 5C). We closely correlate this regulation with the restraint recombination and speculate that that it provides one of several roles by which ATR maintains genome stability.

## MATERIALS AND METHODS

### Yeast strains and molecular biology

The *RuraR* locus (Lambert et al., 2005) plus the *uraR* and *Rura* loci (Lambert et al., 2010) used in this study have been described previously, and the analysis of recombination outcomes was performed as described previously. Checkpoint deletions and alleles for the tagged proteins used were created and introduced by using standard molecular and genetic techniques (Bähler et al., 1998; Moreno et al., 1991; Watson et al., 2008). The strains used are listed in supplementary material Table S2. The *ade6-M375 int::pUC8/his3^+^/RTS1(A2)/ade6-L469* locus was a gift from Matthew Whitby (Ahn et al., 2005). All strains were grown in 30 µM thiamine where indicated. The origins near *RTS1* were renamed as *ori3006/7*. We have previously used *ars3003/4* (Miyabe et al., 2011) and *ars3004/5* (Lambert and Carr, 2005; Lambert et al., 2010; Mizuno et al., 2009; Mizuno et al., 2013).

### Two-dimensional gel electrophoresis

Replication intermediates were analysed and quantified by 2DGE, as reported previously (Lambert et al., 2010). Briefly, zymolyase-treated cells were embedded in an agarose plug, treated with proteinase K and washed several times in Tris-EDTA. After restriction digestion by *Ase*I, replication intermediates were enriched on benzoylated naphthoylated DEAE (BND)–cellulose columns, precipitated and separated by 2DGE using 0.35% and 0.9% agarose for the first and second dimensions, respectively. Quantification of replication intermediates was performed using a phosphor-imager (Typhoon-trio) to detect ^32^P signal. Briefly, fork-termination and joint-molecules signals were quantified as the percentage of stalled fork signal. Chromosomal rearrangements were analysed by pulsed-field gel electrophoresis or Southern blotting as reported previously (Lambert et al., 2010; Lambert et al., 2005).

### Visualisation of tagged proteins by ChIP

ChIP was performed as described previously (Lambert et al., 2005), but with sonication performed with a Diagenode Bioruptor at high setting (7 cycles; 30 seconds on +30 seconds off) to achieve a fragment size of 200–300 bp. The distance of primer pairs away from the *RTS1* RFB is indicated on Fig. 2A and Fig. 2C. The primer sequences are given in supplementary material Table S1. Enrichment was normalised to an internal control (*ade6* locus). Anti-GFP (rabbit polyclonal, Invitrogen), anti-Rpa2 (also known as Ssb2) [rabbit polyclonal; a gift from Hisao Masukata (Osaka University, Osaka, Japan)] or anti-human-Rad51 (H-92 rabbit polyclonal, Santa Cruz Biotechnology) were used at 1∶300, 1∶500 or 1∶100, respectively. Immunocomplexes were precipitated with Protein G Dynabeads (Invitrogen). For each ChIP experiment, wild-type and mutated strains were analysed in parallel. The data (except those shown in Fig. 1D) represent the relative enrichment of immunoprecipitated proteins in a given mutant relative to the enrichment observed in the corresponding wild-type control strain, either when the *RTS1*-RFB is active (arrest ‘on’) or inactive (arrest ‘off’).

### Direct repeat recombination assay

Red colonies (*ade*^−^ cells) were picked from agar plates containing low adenine and no histidine, and were inoculated into 10 ml of rich medium, followed by overnight incubation. Cells were washed and split into two cultures each of 10 ml of Edinburgh Minimal Media (EMM) containing excess adenine and histidine, with or without thiamine. After 48 hours of logarithmic growth, cells from each culture were plated onto YE agar plates containing excess guanine. Cells were concurrently plated onto non-selective medium to determine the number of viable cells. After growth for 3 days, colonies from the plates lacking adenine were counted, to calculate the frequency of *ade^+^* recombinants. Each experiment represents a median of 11 individual plates, and statistical significance was calculated by using Student's *t*-test.

### Replication slippage assay

Replication slippage was scored using the reporter allele *ura4-sd20*, which contains a duplication of 20 nt flanked by 5 nt of microhomology, as described previously (Iraqui et al., 2012). DNA synthesis associated with homologous-recombination-dependent fork restart is error prone, liable to replication slippage leading to the restoration of a functional *ura4^+^* gene and, thus, an induction of ura^+^ colonies. Several single 5-FOA^R^ colonies were grown independently on uracil-containing plates with or without thiamine for 2–3 days, and then inoculated into uracil-containing medium with or without thiamine for 2 days at 30°C. Appropriate dilutions were plated onto supplemented minimal medium and uracil-free plates. Colonies were counted after incubation at 30°C for 5–7 days, and the frequency of ura^+^ colonies was determined. Statistical significance was determined using the nonparametric Mann-Whitney U test.

## Supplementary Material

Supplementary Material
