# Supplementary material for: The extent of error-prone replication restart by homologous recombination is controlled by Exo1 and checkpoint proteins
Source: J Cell Sci. 2014 Jul 1;127(13):2983–94. doi: 10.1242/jcs.152678 (PMC4075360; doi:10.1242/jcs.152678)
Supplement: Supplementary Material [file supp_127_13_2983__index.html]

The extent of error-prone replication restart by homologous recombination is controlled by Exo1 and checkpoint proteins — Supplementary Material 

# The extent of error-prone replication restart by homologous recombination is controlled by Exo1 and checkpoint proteins

## JCS152678 Supplementary Material

**Files in this Data Supplement:**

- **Supplementary Material**
