# Supplementary material for: The extent of error-prone replication restart by homologous recombination is controlled by Exo1 and checkpoint proteins
Source: J Cell Sci. 2014 Jul 1;127(13):2983–94. doi: 10.1242/jcs.152678 (PMC4075360; doi:10.1242/jcs.152678)
Supplement: Supplementary Material [file supp_127.13.2983_JCS152678.pdf]

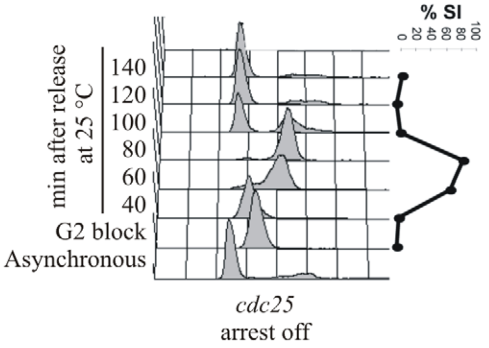

Fig. S1. Activation of the *RTS1*-RFB does induce cell cycle delay. Strain containing the *RuraR* locus and harboring the *cdc25-22* allele were grown at 25°C in minimal media with (arrest off) or without thiamine (arrest on) for 24 hours, then synchronized in G2 at 36°C for 4 hours and released at 25°C. Samples was taken at the indicated times and analyzed for septation index (an S-phase marker) and DNA content by FACS.

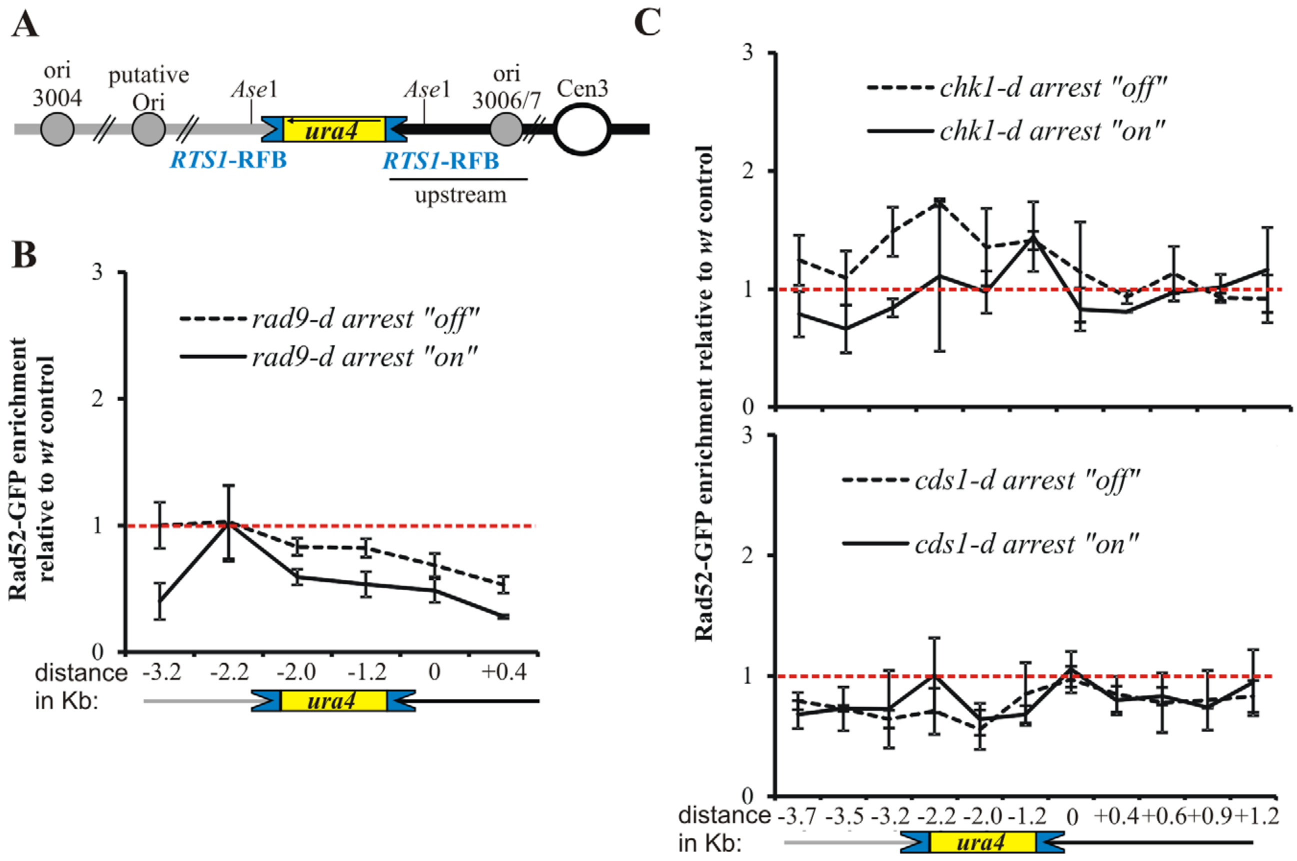

Fig. S2. The checkpoint kinases Cds1 and Chk1 and the nuclease activity of Mre11 are not required to regulate Rad52 recruitment to the *RuraR* locus. (A) Schematic representation of the *RuraR* locus. Grey and black lines indicate telomere and centromere proximal side of the *ura4* gene, respectively. Blue boxes represent the *RTS1*-RFBs and their polarity. *Ase*I sites are ~1 kb *cen* proximal and 0.6 Kb *tel* proximal from RTS1 (see Fig. 1A for further details). (B). Rad52-GFP enrichment relative to wt at the RuraR locus in the rad9-d strains, as described for Fig. 1D. ChIP of Rad52-GFP followed by qPCR was carried out on the indicated *RuraR rad52*$^{+}$*-GFP* strains after 40 hours growth either with or without thiamine (arrest "off" and "on" respectively). Cells containing the *RuraR* locus in a wild type (*wt*) background were analysed alongside an isogenic strain harboring the *rad9-d* alleles. Values are the mean from two to three independent experiments, with ± 95% CI. (C). An equivalent experiment for the *chk1 day* (top panel) and *cds1 day* (bottom panel) strain.

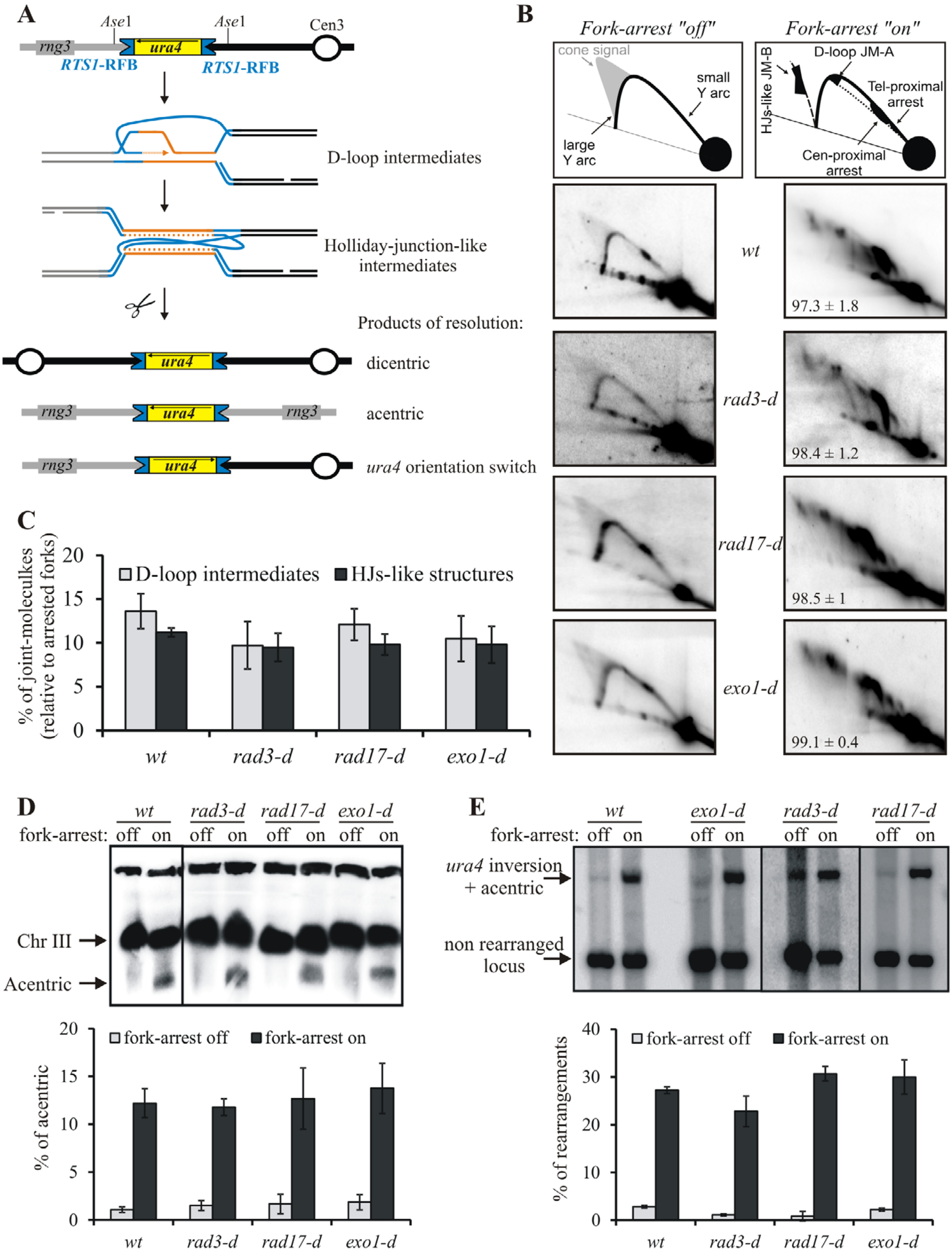
A
Ase1
Ase1
Cen3
rng3
ura4
RTS1-RFB
RTS1-RFB
D-loop intermediates
Holliday-junction-like intermediates
Products of resolution:
dicentric
acentric
ura4 orientation switch
B
Fork-arrest "off"
Fork-arrest "on"
cone signal
small Y arc
large Y arc
D-loop JM-A
HJs-like JM-B
Tel-proximal arrest
Cen-proximal arrest
wt
rad3-d
rad17-d
exo1-d
97.3 ± 1.8
98.4 ± 1.2
98.5 ± 1
99.1 ± 0.4
C
% of joint-moleculkes (relative to arrested forks)
D-loop intermediates
HJs-like structures
D
fork-arrest: off on off on off on off on
Chr III
Acentric
% of acentric
fork-arrest off
fork-arrest on
E
ura4 inversion + acentric
non rearranged locus
% of rearrangements

Fig. S3. Checkpoint pathways do not affect template exchange occurring during the restarting of the fork at *RuraR*. (A) Diagram of the *RuraR* locus (see Fig. 1 for details). Upon fork-arrest at the *RTS1*-RFB stalled nascent strands switch template and invade the opposite *RTS1* sequence, leading to the formation of an early joint-molecule (D-loop). The incoming of the opposite fork leads to a reciprocal template switch of stalled nascent strands leading to the formation of a late joint-molecule containing Holliday junctions (HJs). The resolution of HJ-like structures leads to three distinct products: acentric or dicentric isochromosomes and the inversion of *ura4* orientation. (B). Analysis of RIs by 2DGE from indicated strains grown in media containing or lacking thiamine (fork-arrest "off" and "on" respectively). Top panels are diagrams of RIs within the A*se*1 restriction fragment analysed by 2DGE in indicated conditions. Numbers indicate the percentage of forks arrested by the *RTS1*-RFB ± standard deviation (SD). The panels from *wt* and *rad3-d* strains are also presented on Fig. 1C. (C). Quantification of joint-molecules observed in Panel B. Values are the mean of three independent experiments ± s.d. (D). Top panel: chromosomes from indicated strains and conditions were separated by Pulse Field Gel Electrophoresis (PFGE) and analysed by Southern-blotting using an *rng3* probe, located *telomere*-proximal from *ura4*. Cells were grown for 48 hours in media with (fork arrest "off") or without thiamine (fork-arrest "on"). Bottom panel: Quantification of the acentric level in indicated strains and conditions. Values correspond to the mean of three independent experiments ± s.d. (E). Top panel: representative Southern blot showing accumulation of rearrangement following digestion of genomic DNA with *Eco*RV, Southern blotting and hybridisation with a *ura4* probe. Cells from indicated strains were grown for 48 hours with (fork-arrest "off") or without (fork-arrest "on") thiamine. Bottom panel: quantification of the amount of chromosomal rearrangements observed in the top panel. Values correspond to the mean of three independent experiments ± s.d.

Supplementary Table 1. qPCR primers used for ChIP

Primer	Sequence (5′-3′)	Size	Primer	Sequence (5′-3′)	Size
R1400F	CACCAAAGCCGATTTATTCC	118	L600R	CCCTGGCGGTTGTAGTTAGT	
R1400R	CCCGTATTTCTCAAAATAAATCTCA		L900F	AACGGTTGTAGAAGACGAGCA	112
R1200F	AGTATGTTAAGATGGCTTGTCTGTTC	80	L900R	TGTAAGCACACCTTCAATGTATCA	
R1200R	CCTAATGACAGCGGCAAGA		L1200F	CAAATATGGAGTGCTTTATCTCCA	87
R1000F	TTGCTTATCGATCACTGAATATTTCT	94	L1200R	CCTTTAATGAACGAAACTTAGAATCA	
R1000R	AAGATGCATTTCCATTATAATCCTCTA		L1400F	AACATCGGTGACCTCGTTCT	83
R800F	TCTGATTTAAGGATACGTAGAACTGC	81	L1400R	CTCTTCGCTCCAAGCGTTAT	
R800R	TGCTACTTATACAACCCGTGTATTG		L1600F	CGACATAGATTCCGCTGATG	115
R600F	ACATGTGACATATAGTGATACACAACG	87	L1600R	TTCATCATTGTCGGTGTTCC	
R600R	GGGTACGAACTTACAGTGTTTACCAA		L1800F	GGCAAAGTAGATCCGACAGC	101
R400F	CACACTTGCTCTGTACACGTATTCT	115	L1800R	TGAATACGCCGTTACTCCTAAAG	
R400R	AGGATCCATGATGCACAGATT		L2000F	GGTGACGTCAAATCACCAAA	84
R5F	TTGCCAAACATCCTCCTACC	88	L2000R	CCCACAAAGCTCTTCATACAAA	
R5R	GAAACACAAGCCAAAGTTGC		L2200F	AAGGCAAGAAACGCTGAGAC	106
R3F	TTCTGTTCCAACACCAATGTTT	97	L2200R	GGCATGCATACTACCCGATAA	
R3R	TGTACAAAGCCAATGAAAGATG		L2400F	TTGCTTTCAAGAAAGTCACTGTAGTT	82
uraF	TGATATGAGCCCAAGAAGCA	80	L2400R	GTGCAGGAAGTGATGAATCG	
uraR	CAAATTCGCAGACATTGGAA		L2600F	ACAGATAAATGGCGCAAACC	109
L5F	AGGGCATTAAGGCTTATTTACAGA	118	L2600R	CGAACACAAGTGTGGTCTTTG	
L5R	TCACGTTTAATTTCAAACATCCA		L2800F	GCATCAATTGCTCGTCAAAA	116
L3F	TTTAAATCAAATCTTCCATGCG	91	L2800R	GCGGTAAAATCGTGATATTTGAG	
L3R	TGTACCCATGAGCAAACTGC		L3000F	TCGTACGTACTGCATCGCTATAA	115
L400F	ATCTGACATGGCATTCCTCA	101	L3000R	TTCATCCTACCTTCTAGTTCGGTAA	
L400R	GATGCCAGACCGTAATGACA		Ade623	GGCTGCCTCTACCATCATTC	109
L600F	CCATTGACTAGGAGGACTTTGAG	86	Ade625	TTAAGCTGAGCTGCCAAGGT	

Size = amplicon size

Supplementary Table 2. Strains used in the study.

Strains	Genotype	Reference
ETS17	h^- smt0 sup35:nmt41:$rtf1^+$ ade6-704 leu1-32 RuraR :$ura4^+$	Lambert 2005
ETS20	h^- smt0 sup35:nmt41:$rtf1^+$ ade6-704 leu1-32 $ura4^+$	Lambert 2005
ETS24/SL895	h^- smt0 sup35:nmt41:$rtf1^+$ ade6-704 leu1-32 RuraR :$ura4^+$rad3 ::HygMX	This study
ETS28/SL894	h^- smt0 sup35:nmt41:$rtf1^+$ ade6-704 leu1-32 RuraR :$ura4^+$rad9 ::NATMX	This study
ETS32 /SL896	h^- smt0 sup35:nmt41:$rtf1^+$ ade6-704 leu1-32 RuraR :$ura4^+$rad17 ::HygMX	This study
ETS207	h^- smt0 sup35:nmt41:$rtf1^+$ ade6-704 leu1-32 RuraR :$ura4^+$rad50::KanMX	Lambert 2005
ETS44	h^- smt0 sup35:nmt41:$rtf1^+$ ade6-704 leu1-32 RuraR :$ura4^+$chk1::KanMX	This study
ETS52	h^- smt0 sup35:nmt41:$rtf1^+$ ade6-704 leu1-32 RuraR :$ura4^+$cds1::NATMX	This study
ETS193/SL893	h^- smt0 sup35:nmt41:$rtf1^+$ ade6-704 leu1-32 RuraR :$ura4^+$exo1::NATMX	This study
SL350	h^- sup35:nmt41:$rtf1^+$ ade6-704 leu1-32 uraR :$ura4^+$	Lambert 2005
SL903/ETS412	h^- smt0 sup35:nmt41:$rtf1^+$ ade6-704 leu1-32 uraR :$ura4^+$ rad17 ::HygMX	This study
SL907/ETS410	h^- smt0 sup35:nmt41:$rtf1^+$ ade6-704 leu1-32 uraR :$ura4^+$ rad3 ::HygMX	This study
SL916/JZ52	h^- smt0 sup35:nmt41:$rtf1^+$ ade6-704 leu1-32 uraR :$ura4^+$ exo1::NATMX	This study
II306/JZ54	h^- smt0 sup35:nmt41:$rtf1^+$ ade6-704 leu1-32 uraR :$ura4^+$ exo1::NATMX rad3 ::HygMX	This study
JZ107	h^- smt0 sup35:nmt41:$rtf1^+$ ade6-704 leu1-32 uraR :$ura4^+$ exo1::NATMX rad17::HygMX	This study
ETS57	h^- smt0 sup35:nmt41:$rtf1^+$ ade6-704 leu1-32 RuraR :$ura4^+$ $rad52^+$-GFP :KanMX	This study
ETS62	h^- smt0 sup35:nmt41:$rtf1^+$ ade6-704 leu1-32 RuraR :$ura4^+$ $rad52^+$-GFP :KanMX rad3 ::HygMX	This study
ETS66	h^- smt0 sup35:nmt41:$rtf1^+$ ade6-704 leu1-32 RuraR :$ura4^+$ $rad52^+$-GFP :KanMX rad17 ::HygMX	This study
ETS70	h^- smt0 sup35:nmt41:$rtf1^+$ ade6-704 leu1-32 RuraR :$ura4^+$ $rad52^+$-GFP :KanMX chk1::KanMX	This study
ETS77	h^- smt0 sup35:nmt41:$rtf1^+$ ade6-704 leu1-32 RuraR :$ura4^+$ $rad52^+$-GFP :KanMX cds1::KanMX	This study
ETS136	h^- smt0 sup35:nmt41:$rtf1^+$ ade6-704 leu1-32 RuraR :$ura4^+$ $rad52^+$-GFP :KanMX rad9 ::NATMX	This study
ETS185	h^- smt0 sup35:nmt41:$rtf1^+$ ade6-704 leu1-32 RuraR :$ura4^+$ $rad52^+$-GFP :KanMX rad3 ::HygMX rad17 ::NATMX	This study
ETS233	h^- smt0 sup35:nmt41:$rtf1^+$ ade6-704 leu1-32 RuraR :$ura4^+$ $rad52^+$-GFP :KanMX exo1 ::NATMX	This study
ETS235	h^- smt0 sup35:nmt41:$rtf1^+$ ade6-704 leu1-32 RuraR :$ura4^+$ $rad52^+$-GFP :KanMX exo1 ::NATMX rad3 ::HygMX	This study
ETS241	h^- smt0 sup35:nmt41:$rtf1^+$ ade6-704 leu1-32 RuraR :$ura4^+$ $rad52^+$-GFP :HygMX rad50 ::KanMX	This study
ETS254	h^- smt0 sup35:nmt41:$rtf1^+$ ade6-704 leu1-32 RuraR :$ura4^+$ $rad52^+$-GFP :HygMX rad3::NATMX	This study
ETS320	h^- smt0 sup35:nmt41:$rtf1^+$ ade6-704 leu1-32 RuraR :$ura4^+$ $rad52^+$-GFP :HygMX rad3 ::NatMX rad50::KanMX	This study
ETS375	h^- smt0 sup35:nmt41:$rtf1^+$ ade6-704 leu1-32 RuraR :$ura4^+$ $rad52^+$-GFP :KanMX rad32-D65N	This study
ETS383	h^- smt0 sup35:nmt41:$rtf1^+$ ade6-704 leu1-32 RuraR :$ura4^+$ $rad52^+$-GFP :KanMX rad3::HygMX rad32-D65N	This study
YC5	h^- sup35:nmt41:$rtf1^+$ ade6-704 leu1-32 ura4-sd20	Iraqui 2012
YC13	h^- sup35:nmt41:$rtf1^+$ ade6-704 leu1-32 ura4-sd20 :RTS1	Iraqui 2012
YC21	h^- sup35:nmt41:$rtf1^+$ ade6-704 leu1-32 RTS1 :ura4-sd20	Iraqui 2012
II264	h^- sup35:nmt41:$rtf1^+$ ade6-704 leu1-32 ura4-sd20 exo1 :NATMX	This study
II258	h^- sup35:nmt41:$rtf1^+$ ade6-704 leu1-32 ura4-sd20 :RTS1 exo1 :NATMX	This study
II324	h^- sup35:nmt41:$rtf1^+$ ade6-704 leu1-32 RTS1 :ura4-sd20 exo1 :NATMX	This study
II340	h^- sup35:nmt41:$rtf1^+$ ade6-704 leu1-32 ura4-sd20 rad3 ::HygMX	This study
II317	h^- sup35:nmt41:$rtf1^+$ ade6-704 leu1-32 ura4-sd20 :RTS1 rad3 ::HygMX	This study
II334	h^- sup35:nmt41:$rtf1^+$ ade6-704 leu1-32 RTS1 :ura4-sd20 rad3 ::HygMX	This study
II359	h^- sup35:nmt41:$rtf1^+$ ade6-704 leu1-32 ura4-sd20 rad3 ::HygMX exo1 :NATMX	This study
II352	h^- sup35:nmt41:$rtf1^+$ ade6-704 leu1-32 ura4-sd20 :RTS1 rad3 ::HygMX exo1 :NATMX	This study
II346	h^- sup35:nmt41:$rtf1^+$ ade6-704 leu1-32 RTS1 :ura4-sd20 rad3 ::HygMX exo1 :NATMX	This study
ETS349	h^- smt-0 ura4-D18 leu1-32 his3-D1 arg3-D4 ade6-M375 int::pUC8/his3+/RTS1(A2)/ade6-L469 tRNA:nmt41:rtf1+	This study
IM836	h^- smt-0 ura4-D18 leu1-32 his3-D1 arg3-D4 ade6-M375 int::pUC8/his3+/RTS1(A2)/ade6-L469 tRNA:nmt41:rtf1+ rad3::HygMX	This study
IM838	h^- smt-0 ura4-D18 leu1-32 his3-D1 arg3-D4 ade6-M375 int::pUC8/his3+/RTS1(A2)/ade6-L469 tRNA:nmt41:rtf1+ rad17::HygMX	This study
IM840	h^- smt-0 ura4-D18 leu1-32 his3-D1 arg3-D4 ade6-M375 int::pUC8/his3+/RTS1(A2)/ade6-L469 tRNA:nmt41:rtf1+ exo1::NATMX	This study
IM842	h^- smt-0 ura4-D18 leu1-32 his3-D1 arg3-D4 ade6-M375 int::pUC8/his3+/RTS1(A2)/ade6-L469 tRNA:nmt41:rtf1+ rad3::HygMX exo1::NATMX	This study
IM844	h^- smt-0 ura4-D18 leu1-32 his3-D1 arg3-D4 ade6-M375 int::pUC8/his3+/RTS1(A2)/ade6-L469 tRNA:nmt41:rtf1+ rad17::HygMX exo1::NATMX	This study
